# Characteristics of DNA-AuNP networks on cell membranes and real-time movies for viral infection

**DOI:** 10.1016/j.dib.2015.12.044

**Published:** 2016-01-13

**Authors:** Chunmei Li, Linling Zheng, Xiaoxi Yang, Xiaoyan Wan, Wenbi Wu, Shujun Zhen, Yuanfang Li, Lingfei Luo, Chengzhi Huang

**Affiliations:** a Key Laboratory of Luminescent and Real-Time Analytical Chemistry (Southwest University), Ministry of Education, College of Pharmaceutical Sciences, Southwest University, Chongqing 400715, PR China; b Chongqing Key Laboratory of Biomedical Analysis (Southwest University), Chongqing Science & Technology Commission, College of Chemistry and Chemical Engineering, Southwest University, Chongqing 400715, PR China; c College of Life Sciences, Southwest University, Chongqing 400715, PR China

**Keywords:** DNA-AuNP networks, Cell membrane, Viral infection, Inhibition

## Abstract

This data article provides complementary data for the article entitled “DNA-AuNP networks on cell membranes as a protective barrier to inhibit viral attachment, entry and budding” Li et al. (2016) [1]. The experimental methods for the preparation and characterization of DNA-conjugated nanoparticle networks on cell membranes were described. Confocal fluorescence images, agarose gel electrophoresis images and hydrodynamic diameter of DNA-conjugated gold nanoparticle (DNA-AuNP) networks were presented. In addition, we have prepared QDs-labeled RSV (QDs-RSV) to real-time monitor the RSV infection on HEp-2 cells in the absence and presence of DNA-AuNP networks. Finally, the cell viability of HEp-2 cells coated by six types of DNA-nanoparticle networks was determined after RSV infection.

**Specifications table**

**Table:** 

Subject area	Chemistry
More specific subject area	Biomaterials, anti-viral research
Type of data	Text file, image (microscopy, etc), graph, figure, movie
How data was acquired	Mass spectroscopy (Bruker Daltonics flexAnalysis, USA), UV–vis absorption spectra (Hitachi U-3010 spectrophotometer, Tokyo, Japan), DLS and zeta potential (Zetasizer Nano-ZS System, Malvern Inc), Fluorescence Microscope (Olympus IX-81, Tokyo, Japan), SEM (Hitachi S-4800, Tokyo, Japan), Cell viability (Biotek Microplate Reader,USA)
Data format	Analyzed
Experimental factors	Cells with or without coating by DNA-AuNP networks were infected by RSV
Experimental features	Cell membranes were fabricated by DNA-AuNP networks. After virus infection, immunofluorescence imaging and cell viability tests were carried out.
Data source location	Southwest University, Chongqing, PR China
Data accessibility	Data are with this article

**Value of the data**

• This data provides detailed methods for the preparation and characterization of DNA-AuNP networks on cell membrane.
• The data is useful for developing movies to real-time monitor the inhibition of viral infection.
• It provides multiform ways to investigate the stability and antiviral ability of DNA-AuNP networks.
• This data may provide a pathway for the development of broad-spectrum antiviral agents.

## 1 Data

This paper presents data related to the research article entitled “DNA-AuNP networks on cell membranes as a protective barrier to inhibit viral attachment, entry and budding” [1]. The data here include the characterization of DNA-conjugated nanoparticle networks on cell membranes, the stability test of DNA-AuNP networks against enzymatic cleavage, the immunofluorescence images of HEp-2 cells coated by DNA-magnetic nanoparticle (DNA-MNP) networks, the real-time movies for RSV infection, and cell viability of HEp-2 cells fabricated by six types of DNA-nanoparticle networks.

## 2 Experimental design, materials and methods

### 2.1 Chemicals and materials

Streptavidin (SA) was purchased from Beijing Biosynthesis Biotechnology Co. Ltd. (Beijing, China) and bovine serum albumin (BSA) was purchased from Beijing Dingguo Changsheng Biotechnology Co. Ltd. (Beijing, China). Streptavidin-coated iron oxide nanoparticles (SA-MNPs, Ocean Nanotech) of 10 nm and 30 nm diameter were resuspended at 0.1 mg/mL in 100 mM phosphate-buffered saline (PBS). Citrate coated gold nanoparticles (AuNPs) of 13 nm, 30 nm, and 50 nm diameters were prepared by altering the quantity of HAuCl_4_ and reducing agents according to a previously published method [2]. Citrate coated silver nanoparticles (AgNPs) of 30 nm diameter were obtained by reducing AgNO_3_ with trisodium citrate according to the modified Lee–Meisel method [3].

**Fig. 1:**
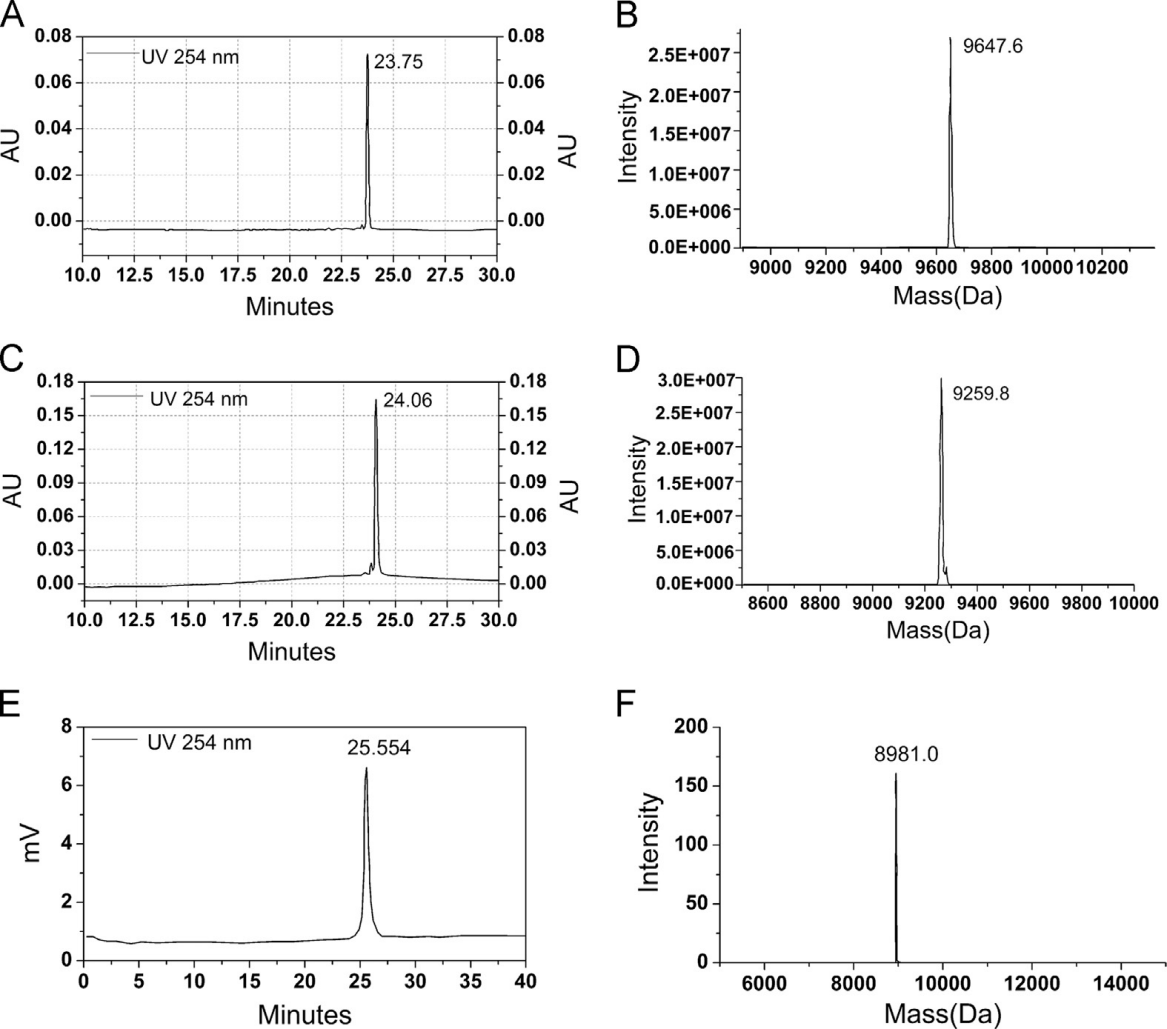
Purification and characterization of biotinylated DNA sequences P1 and P2 and linker DNA sequence. DNA sequences P1 (profile A) and P2 (profile C) were purified by capillary electrophoresis (CE), while the linker DNA sequence (profile E) was purified by high-performance liquid chromatography (HPLC). ESI mass spectrometry analysis was used to characterize the purified P1 (B), P2 (D) and linker DNA (F). P1, Found mass: 9647.6, expected mass: 9649.49; P2, Found mass: 9259.8, expected mass: 9260.05; linker DNA, Found mass: 8981.0, expected mass: 8991.9.

DNA sequences: P1, 5′-AAA GGG TCT GAG GGA TTT TTT TTT TTT -Bio-3′; P2, 5′-Bio-TTT TTT TTT TTT TTT GTC GTG GGT CT-3′; Linker DNA, 5′-TCC CTC AGA CCC TTT (PEG)_4_ AG ACC CAC GAC AAA-3′. All these DNA sequences were purified by capillary electrophoresis (CE) or high-performance liquid chromatography (HPLC), and characterized by ESI mass spectrometry (Fig. 1).

### 2.2 Preparation and characterization of MNP-P1-P2 and AuNP-SA

MNP-P1, MNP-P2, or MNP-P1-P2 were obtained by incubating SA-MNPs of 30 nm (34 nM, 500 μL) with P1, P2, or P1/P2 (100 μM, 100 μL) in PBS buffer (pH 7.4) at 25°C for 1 h with gentle shaking at 120 r/min. Then the solution was ultracentrifuged at 10000 r/min for 5 min three times to remove the free DNA. At last, MNP-P1, MNP-P2, or MNP-P1-P2 were redispersed in PBS buffer (pH 7.4) and stored at 4 °C, respectively. MNP-P1-P2 was further hybridized with linker DNA by mixing MNP-P1-P2 with linker DNA in PBS buffer for 1 h at room temperature. Transmission electron microscope (TEM) images were used to demonstrate the aggregation of MNP-P1-P2 by DNA hybridization with a Hitachi S-4800 scanning electron microscope (Tokyo, Japan) at 20.0 kV.

**Fig. 2:**
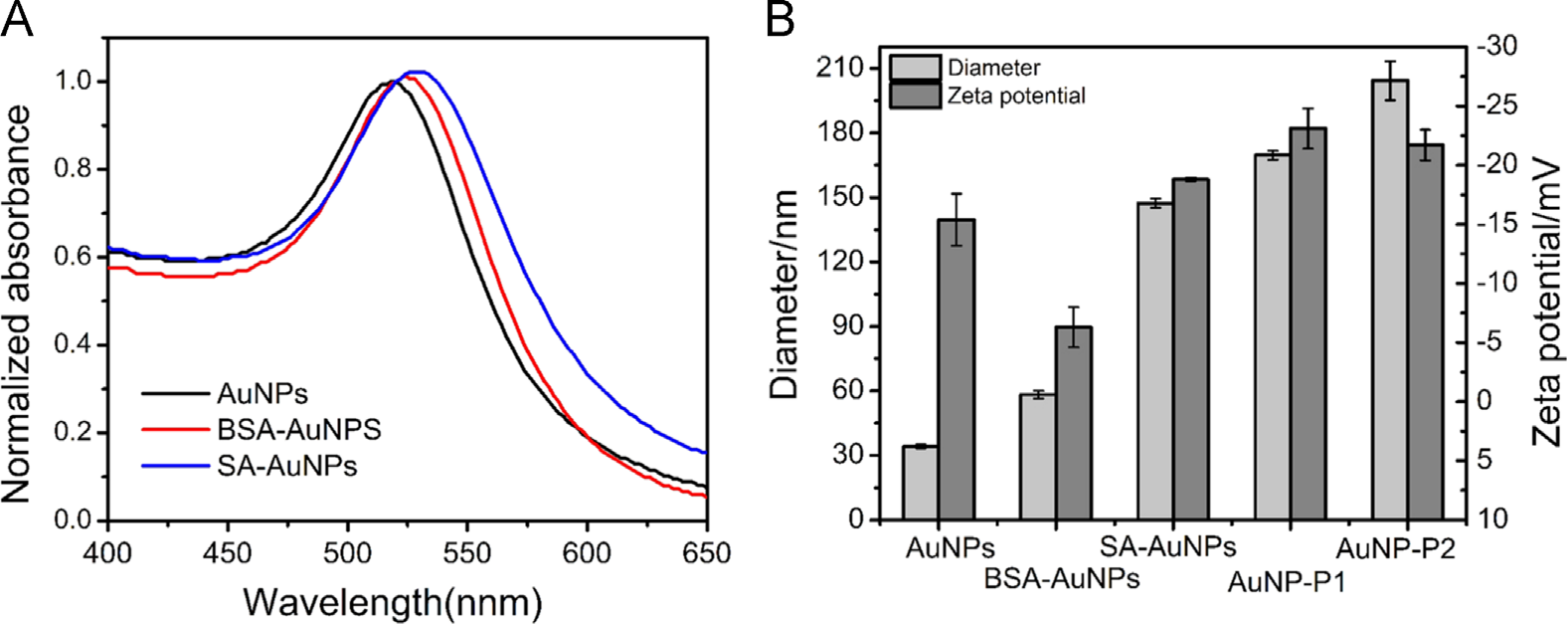
Characterization of BSA-AuNPs and the conjugation with P1 or P2. (A) UV–vis absorption spectra of AuNPs (black line), BSA-AuNPs (red line) and SA-AuNPs (blue line). (B) Hydrodynamic diameter and zeta potential of AuNPs, BSA-AuNPs, SA-AuNPs, AuNP-P1 and AuNP-P2. Error bars represented the standard deviation from three replicated experiments. AuNPs: 0.28 nM. Bio-P1, Bio-P2: 1.0 µM.

SA-AuNPs was prepared by incubating the as-prepared AuNPs of 13 nm (920 μL) with SA (1 mg/mL, 80 μL) for about 30 min at room temperature, followed by the addition of BSA (50 mg/mL, 100 μL) to react another 30 min in order to block the excessive binding sites on AuNPs surface. AuNPs modified with BSA only was used as a control (BSA-AuNPs). SA-AuNPs was further conjugated with P1 or P2 to obtain AuNP-P1 or AuNP-P2 following the above protocol for the preparation of MNP-P1or MNP-P2. UV–vis absorption spectra of AuNPs, BSA-AuNPs and SA-AuNPs were measured by a U-3010 spectrophotometer (Hitachi, Tokyo, Japan) (Fig. 2A). The hydrodynamic diameters and zeta potential of nanoparticles were determined by Zetasizer Nano-ZS System (Malvern Inc) (Fig. 2B).

### 2.3 Characterization of DNA-nanoparticle networks on cell membranes

Scanning electron microscope (SEM) images of HEp-2 cells anchored by DNA-AuNP networks were obtained using a gradient ethanol dehydration method. Accordingly, HEp-2 cells were seeded and cultured on 24-well plates with cover glass slides for about 24 h to reach 50% confluence. After fabrication of DNA-AuNP networks on cell membranes, cells were first fixed by 500 µL of 2.5% glutaraldehyde at room temperature for 2 h, followed by a gradient series of ethanol dehydration from 30%, 50%, 75%, 80%, 95%, to 100%. Each step was maintained for 15 min to ensure complete ethanol saturation throughout the cells. The samples were then sputter-coated with gold for 30 s, and observed using a Hitachi S-4800 scanning electron microscope (Tokyo, Japan) at an operating voltage of 20.0 kV.

Dark-field light scattering imaging of HEp-2 cells was used to demonstrate the formation of DNA-AuNP networks on cell membranes. HEp-2 cells were seeded and cultured on 24-well plates with cover glass slides for about 24 h to reach 50% confluence. After PBS washing, cells were anchored by SA-AuNPs, BSA-AuNPs, AuNP-P1-P2 and DNA-AuNP networks on cell membranes, respectively. Then cells were fixed with 4% paraformaldehyde, sealed with glycerin, and then transferred for dark-field light scattering imaging under an Olympus BX-51 System microscope (Tokyo, Japan) with an Olympus E-510 digital camera (Tokyo, Japan) [2].

**Fig. 3:**
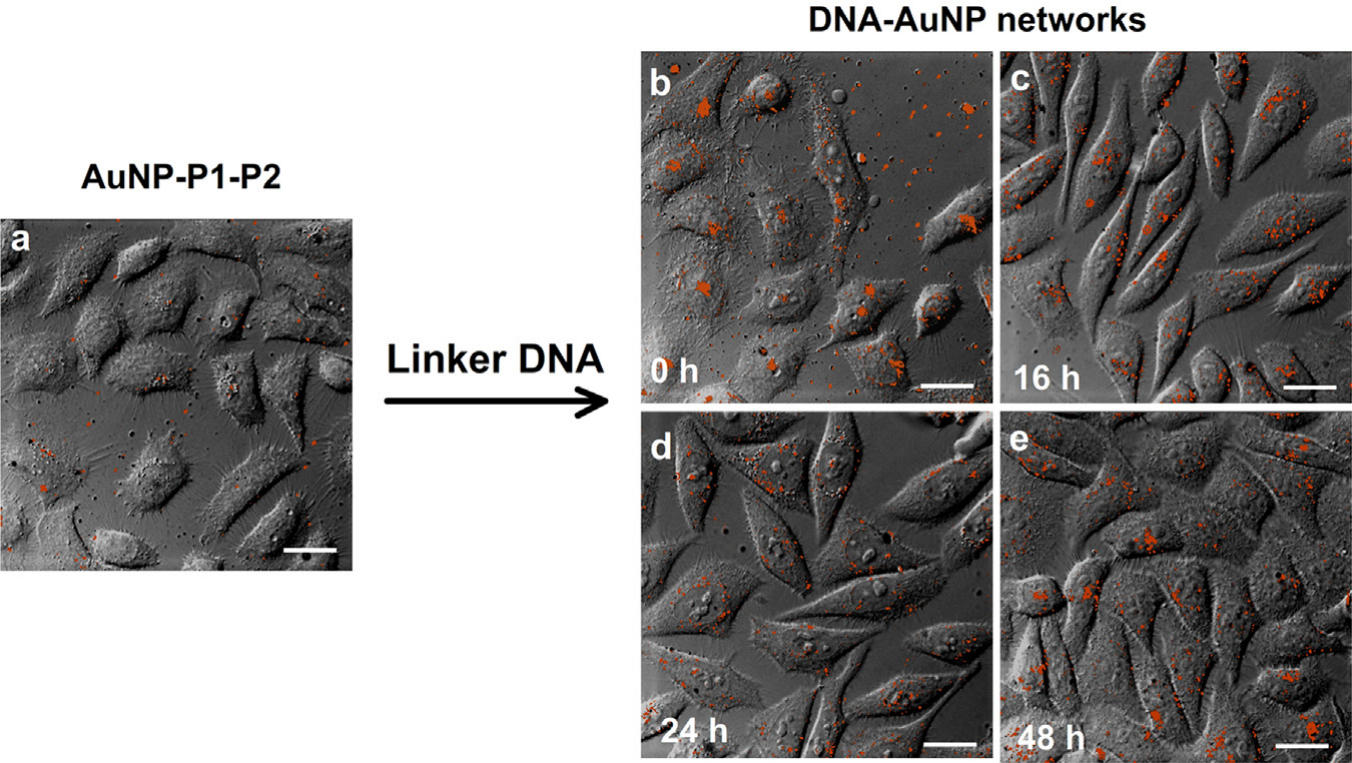
Confocal fluorescence images of biotinylated HEp-2 cells anchored with AuNP-P1-P2 in the absence and presence of linker DNA for incubation at 37 °C for different time. (a) Cells were anchored with AuNP-P1-P2 on cell membranes. (b), (c), (d) and (e) represent cells anchored with DNA-AuNP networks and then incubated at 37 °C for 0 h, 16 h, 24 h and 48 h, respectively. The red fluorescence emission is from Cy3 channel excited with a 530–550 nm laser. HEp-2 cells: 1.0×10^5^ cells mL^−1^. AuNPs: 0.28 nM. Bio-P1, Bio-P2 and linker DNA: 1.0 µM. Scale bar, 20 μm.

Confocal fluorescence images of HEp-2 cells anchored by DNA-AuNP networks were carried out. The fluorescent dye of Cy3 was modified at the 5′ end of oligonucleotide P1. Briefly, HEp-2 cells (1.0×10^5^ cells mL^−1^) were cultured in the 35 mm glass-bottom cell culture dishes (NEST. Corp) over 24 h. After fabrication of DNA-AuNP networks on cell membranes, cells were further incubated at 37 °C for 0 h, 16 h, 24 h and 48 h, respectively (Fig. 3). After washing by PBS, cells were fixed with 4% paraformaldehyde for 20 min. Fluorescent images were acquired using an Olympus IX-81 inverted microscope equipped with an Olympus IX2-DSU confocal scanning system and a Rolera-MGi EMCCD. Colocalization analysis was performed with Image-Pro Plus software. Fluorescent dye of Cy3 was excited at 530–550 nm and detected with a barrier filter BA575-625 nm.

### 2.4 Stability of DNA-AuNP networks against enzymatic cleavage

**Fig. 4:**
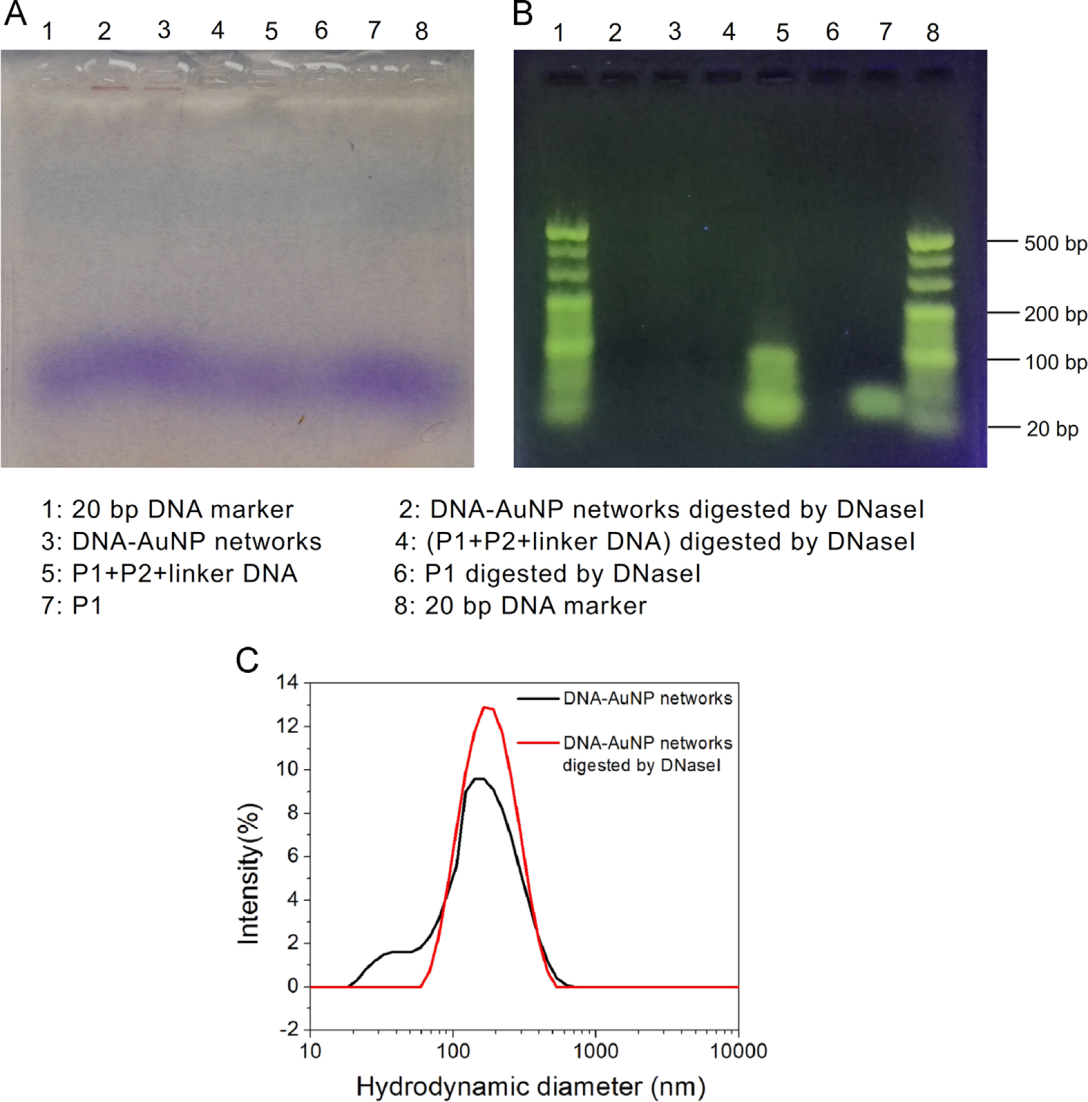
Agarose gel electrophoresis images and hydrodynamic diameter measurement of DNA-AuNP networks after treatment by DNaseI. Samples were first incubated with DNaseI at 37 °C for 20 min. Picture A was taken under the irradiation of visible light. The sample pores in lane 2 and lane 3 showed brick red color, indicating that the DNA-AuNP networks were retained around the sample well. Picture B was taken under the irradiation of UV light. There was no DNA in the lane 6 and lane 4, indicating that P1 and the hybrid of P1+P2+linker DNA were degraded completely by DNaseI. Hydrodynamic diameter measurement showed that neglectable size change was observed for the DNA-AuNP networks after incubation with DNaseI (C).

Agarose gel electrophoresis images were first carried out. At first, DNA sequence P1, the hybrid of P1+P2+linker DNA, and DNA-AuNP networks were incubated with DNaseI at 37 °C for 20 min, respectively. Then 10 μL of reaction products were mixed with 2 μL 6×loading buffer, and then dropped into 2% agarose gel. The gel electrophoresis was run at 120 V for about 50 min in TBE buffer, followed by the Sybr Gold staining (1:2000) at room temperature for 40 min. Finally, gel electrophoresis was photographed with a digital camera under the irradiation of visible light and the UV light, respectively [4] (Fig. 4A and B). In addition, hydrodynamic diameter measurement was used to characterize the size change of the DNA-AuNP networks with or without incubation with DNaseI (Zetasizer Nano-ZS System, Malvern Inc) (Fig. 4C).

### 2.5 Optical microscopic images of biotinylated HEp-2 cells with cytopathogenic effect (CPE)

**Fig. 5:**
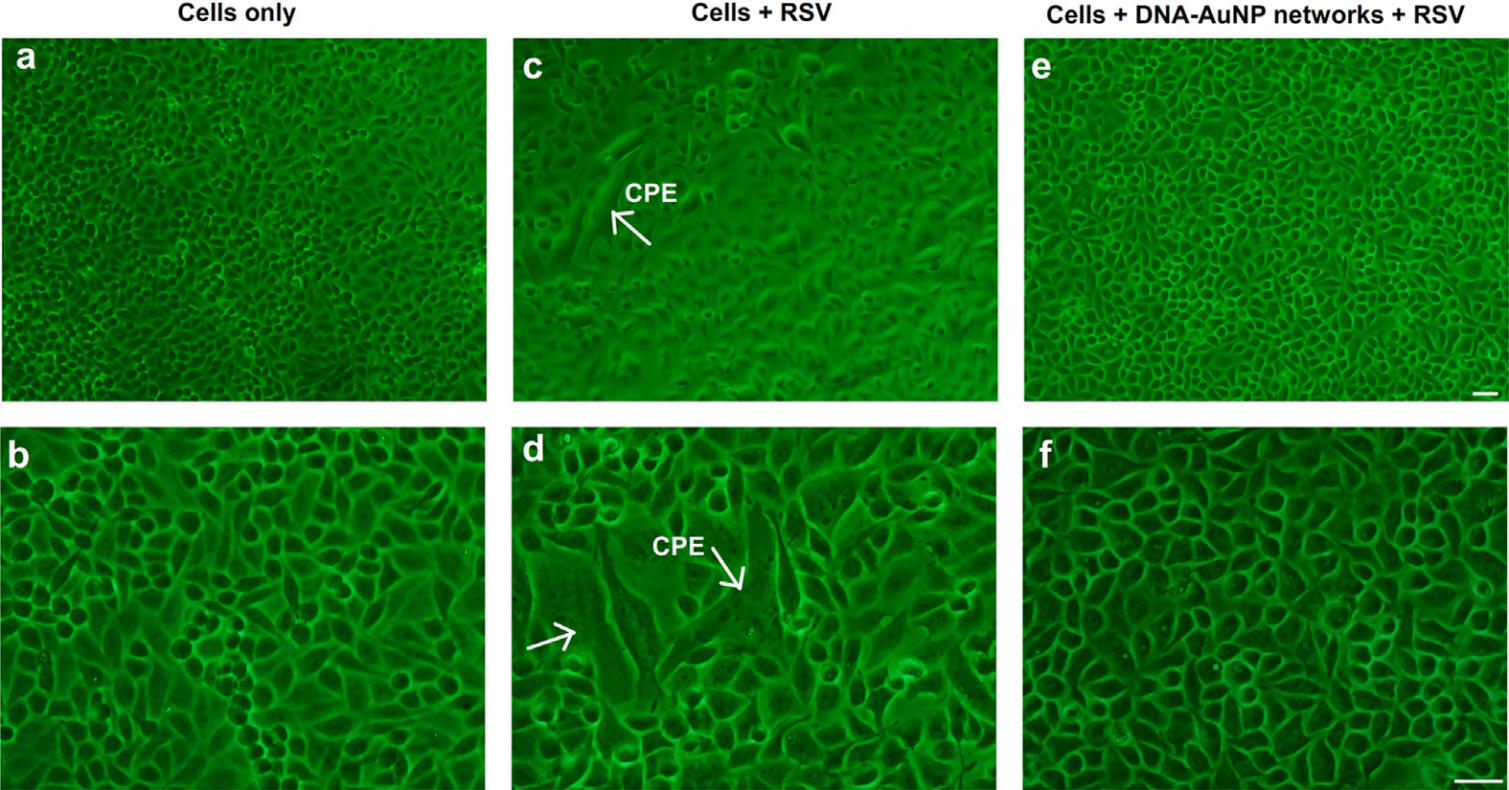
Optical microscopic image of biotinylated HEp-2 cells infected by RSV in the absence and presence of DNA-AuNP networks. (a, b) Biotinylated cells only. Biotinylated cells in the absence (c, d) and presence (e, f) of DNA-AuNP networks were infected with RSV at 4 °C for 30 min. After washing with PBS for two times, cells were further incubated at 37 °C for 2 days. Cytopathogenic effect (CPE) was indicated by the arrows. HEp-2 cells: 1.0×10^5^ cells mL^−1^. AuNPs: 0.28 nM. Bio-P1, Bio-P2 and linker DNA: 1.0 µM. RSV MOI=3. Scale bar, 40 μm.

To visualize the presence of CPE after infection by RSV, optical microscopic images of biotinylated HEp-2 cells infected by RSV in the absence and presence of DNA-AuNP networks were taken after 2 days (Fig. 5). CPE was indicated by the arrows.

### 2.6 Characterization on the formation of DNA-magnetic nanoparticle (DNA-MNP) networks and the inhibition ability on viral attachment

**Fig. 6:**
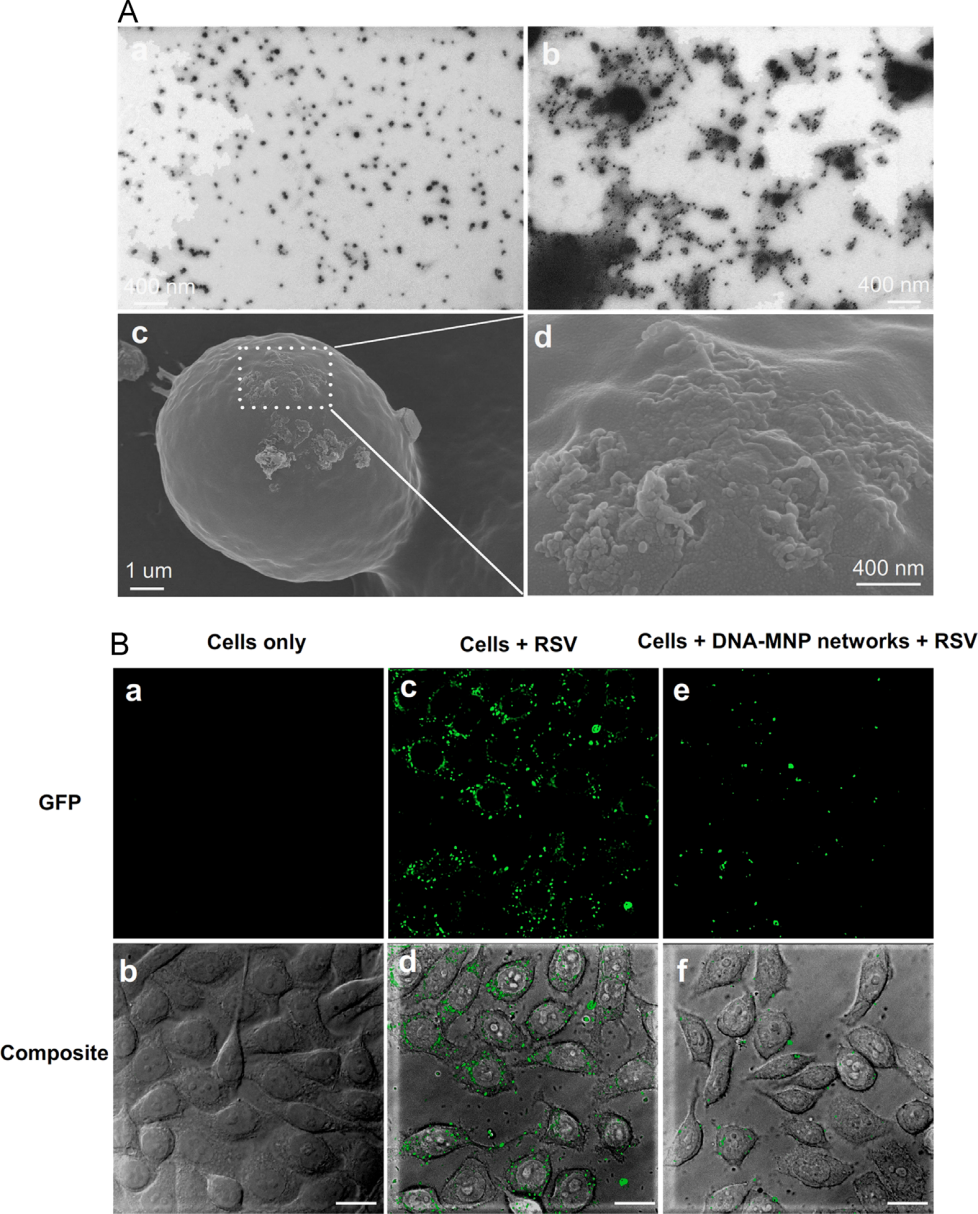
Characterization on the formation of DNA-MNP networks and its inhibition ability of virus attachment on biotinylated HEp-2 cells by immunofluorescence assay. (A) TEM of MNPs-P1-P2 before (a) and after (b) hybridization with linker, and SEM of HEp-2 cells anchored with DNA-MNP networks (c, d). (B) Confocal immunofluorescence images of HEp-2 cells with the inhibition of viral attachment by DNA-MNP networks. Biotinylated cells only (a, b). Biotinylated cells in the absence (c, d) and presence (e, f) of DNA-MNP networks infected with RSV at 4 °C for 30 min. Goat anti-mouse IgG Dylight 488 was used in this immunofluorescence assay. HEp-2 cells: 1.0×10^5^ cells mL^−1^. MNPs: 0.51 nM. Bio-P1, Bio-P2 and linker DNA: 1.0 µM. RSV MOI=3. Scale bar, 20 μm.

**Fig. 7:**
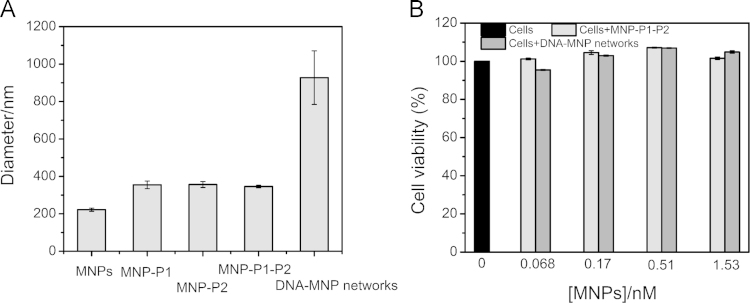
Characterization on the formation of DNA-MNP networks and in vitro cytotoxicity to Biotinylated HEp-2 cells. (A) Hydrodynamic diameter of MNPs, MNP-P1, MNP-P2, MNP-P1-P2 and DNA-MNP networks. (B) in vitro cytotoxicity of MNP-P1-P2 and DNA-MNP networks to HEp-2 cells with different concentrations.

The formation of DNA-MNP networks in solution was characterized by TEM (Fig. 6A ab) and hydrodynamic diameter measurements (Fig. 7A). Then the fabrication of DNA-MNP networks on cell membranes was characterized by SEM using a gradient ethanol dehydration method (Fig. 6A cd). The inhibition ability of DNA-MNP networks on viral attachment was investigated by confocal immunofluorescence images taken after RSV infection at 4 °C for 30 min (Fig. 6B). At last, the in vitro cytotoxicity of DNA-MNP networks to biotinylated HEp-2 cells was tested using the CCK-8 cell viability assay. (Fig. 7B).

**Fig. 8:**
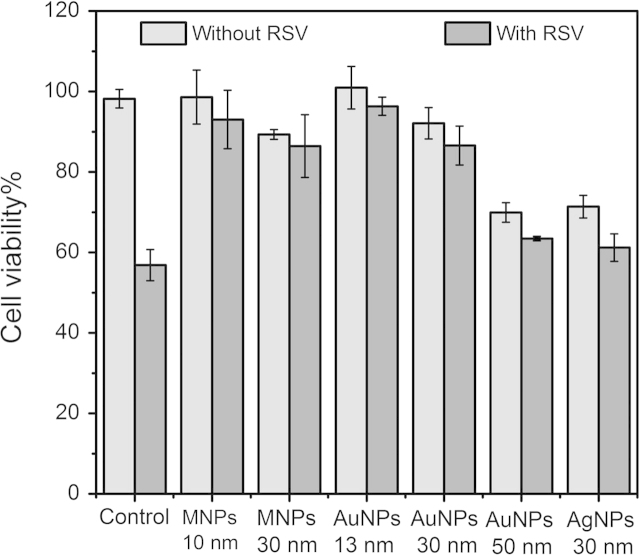
Cell viability of HEp-2 cells pretreated with different kinds of DNA-nanoparticle networks with or without further infection by RSV. MNPs with diameters of 10 nm and 30 nm, AuNPs with diameters of 13 nm, 30 nm, 50 nm, and AgNPs with diameters of 30 nm were used to form DNA-nanoparticle networks, respectively. HEp-2 cells: 1.0×10^5^ cells mL^−1^. MNPs: 0.51 nM. AuNPs: 0.28 nM. AgNPs: 4.6 pM. Bio-P1, Bio-P2 and linker DNA: 1.0 µM. RSV MOI=3.

In addition, six types of DNA-nanoparticle networks with AuNPs (13 nm, 30 nm, and 50 nm), MNPs (10 nm, 30 nm) and silver nanoparticles (AgNPs, 30 nm) were formed on cell membranes, respectively. Then the cell viability of HEp-2 cells at 48 h post-infection was tested (Fig. 8).

### 2.7 Preparation of QDs-labeled virus and real-time monitoring of RSV infection

QDs labelling viruses involves three steps. Firstly, the viruses were concentrated by ultracentrifugation (Beckman Type 70 Ti) at 110,000×*g* for 40 min at 4 °C. After this spin, a virus pellet was clearly visible and the viruses were resuspended in PBS buffer. Subsequently, the concentrated viruses were biotinylated by incubation with 1 mg/mL biotinylation reagent (Sulfo-NHS-LC-LC-Biotin, Thermo Scientific) at 4 °C for 30 min. Unreacted biotinylation reagent was removed with a desalting NAP-5 column. At last, the biotinylated RSV were specifically labeled with QDs through the tight interaction between biotin and streptavidin by incubation with QDs-SA (605 nm, Wuhan Jiayuan Quantum Dots Co.., Ltd.) at 4 °C for 30 min. To remove the free QDs-SA, the reaction solution was loaded onto sucrose cushion (30% sucrose in 0.1 M sodium chloride, 0.01 M Tris–HCl, 0.001 M EDTA, 1 M urea, pH=7.5) and ultracentrifuged (Beckman Type 70 Ti) at 110,000×*g* for 40 min at 4 °C. Purified QDs-labeled RSV (QDs-RSV) were resuspended in PBS buffer and stored at −80°C for further experiments.

The blocking of QDs-RSV attachment to HEp-2 cell membrane by DNA-AuNP networks was real-time monitored by Supplementary movie 1. The inhibition of the entrance and internalization of QDs-RSV into HEp-2 cells by DNA-AuNP networks was subsequently recorded by Supplementary movie 2.

Supplementary material related to this article can be found online at doi:10.1016/j.dib.2015.12.044.

The following is the Supplementary material related to this article Video 1, Video 2.

Video 1 Real-time fluorescence imaging of the attachment of QDs-RSV on biotinylated Hep-2 cells in the absence or presence of DNA-AuNP networks on cell membrane. Movies were taken immediately at room temperature after the addition of QDs-RSV to HEp-2 cells. The red dots represent RSV labeled with QDs (605 nm).

Video 2 Real-time fluorescence imaging of the entrance and internalization of QDs-RSV into biotinylated HEp-2 cells in the absence or presence of DNA-AuNP networks on cell membrane. Movies were taken after the incubation of QDs-RSV with HEp-2 cells at 37 °C for 1 h. The red dots represent RSV labeled with QDs (605 nm).
