# Supplementary figures and images for: Characteristics of DNA-AuNP networks on cell membranes and real-time movies for viral infection
Source: Data Brief. 2016 Jan 13;6:652–60. doi: 10.1016/j.dib.2015.12.044 (PMC4735472; doi:10.1016/j.dib.2015.12.044)

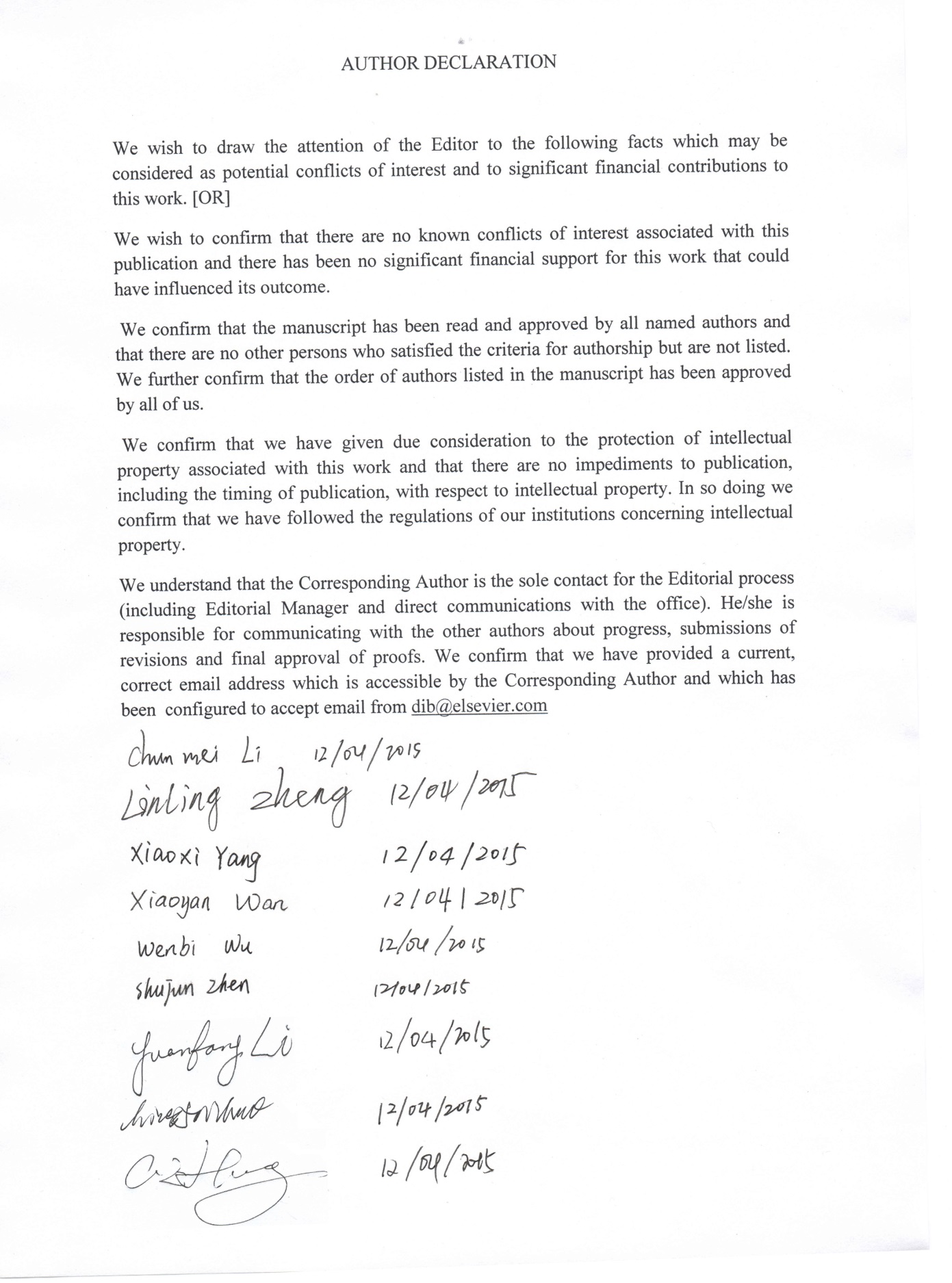

Supplement: Supplementary file 3 — Supplementary material [file mmc3.doc]
